# Characteristics and outcomes of patients with LAM receiving sirolimus in France based on real-life data

**DOI:** 10.3389/fmed.2024.1494713

**Published:** 2025-01-08

**Authors:** Vincent Cottin, Antoine Cases, Virginie Bourdin, Martine Reynaud-Gaubert, Sandrine Hirschi, Mallorie Kerjouan, Rémi Diesler, Brieux Chardès, Stéphane Fiévez, Nada Assi, Aurélie Schmidt, Hélène Denis, Lidwine Wémeau-Stervinou, Yurdagul Uzunhan

**Affiliations:** ^1^Department of Respiratory Medicine, National Reference Centre for Rare Pulmonary Diseases, member of ERN-LUNG, Louis Pradel Hospital, Hospices Civils de Lyon, Lyon, France; ^2^UMR 754, INRAE, Claude Bernard University Lyon 1, Lyon, France; ^3^Pfizer, Paris, France; ^4^Department of Respiratory Medicine, Competence Centre for Rare Pulmonary Diseases, Marseille University Hospital, APHM, Aix Marseille University, Marseille, France; ^5^Department of Respiratory Medicine, Competence Centre for Rare Pulmonary Diseases, Strasbourg University Hospital, Strasbourg, France; ^6^Service de Pneumologie Centre de Compétences pour Les Maladies Pulmonaires Rares, Hôpital Pontchaillou, Rennes, France; ^7^Heva, Lyon, France; ^8^Service de pneumologie centre de référence des maladies pulmonaires rares, Institut Coeur Poumon, CHU de Lille, Lille, France; ^9^Department of Respiratory Medicine, Reference Centre for Rare Pulmonary Diseases, APHP, Hôpital Avicenne, INSERM U 1272, Université Sorbonne Paris-Nord, Bobigny, France

**Keywords:** lymphangioleiomyomatosis, lung-disease, sirolimus, rapamycin, real-life data, survival, tuberous sclerosis complex

## Abstract

LAM is a rare multi-cystic lung disease for which treatment with sirolimus is indicated in cases of moderate or severe lung disease or declining lung function. The aim of this study was to describe patients treated with sirolimus for LAM and their outcomes. This retrospective observational study was based on data from the French national health insurance data system (SNDS). All adult women receiving sirolimus were identified in France between 2014 and 2021. In the absence of a specific LAM code in the system, an algorithm was developed to identify patients treated for possible LAM exclusion of other sirolimus indications (transplantation, graft-versus-host disease), or probable LAM (among possible LAM, patients hospitalized for pneumothorax, pleural drainage, pleurisy, ascites, chronic respiratory failure, lung transplantation, or angiomyolipoma). Over the entire study period, 638 patients were considered as treated with sirolimus for possible LAM, including 208 patients treated for “probable” LAM and 33 patients for TSC-LAM. Median [Q1; Q3] age at index date was 45.0 years [34.0; 58.5] for patients with probable LAM and 40.0 years [28.0; 56.0] for patients with TSC-LAM. Overall, the number of incident patients varied from 28 to 96 each year for possible LAM, from 11 to 33 each year for probable LAM and from 1 to 4 patients each year for TSC-LAM patients. In 2021, the incidence rate of patients treated with sirolimus for probable LAM in France was estimated at 0.9 per 1,000,000 French adult women and the prevalence rate at 6.3 per 1,000,000 French adult women. The 5-year survival after sirolimus initiation was 84% (95% CI: 76%; 90%) for probable LAM patients, and 77% (95% CI: 48%; 91%) for TSC-LAM patients. This study provides an updated epidemiological estimate of LAM patients treated with sirolimus in France between 2014 and 2021. Even though some of the results should be interpreted cautiously in the light of limitations related to the use of claims database, evolution of the disease and missing safety data, the information retrieved in this study is very valuable, as few studies provide real-world information on LAM populations.

## Introduction

Lymphangioleiomyomatosis (LAM) is a rare multicystic, low-grade neoplastic, lung disease. It is characterized by an abnormal proliferation of smooth muscle-like cells (LAM cells) in the lung, especially around the lymphatics, small airways, and vascular tracts. This uncontrolled growth leads to lung cysts that affect the respiratory function, as well as the spread of cells to extrapulmonary organs, such as in angiomyolipomas and lymphangioleiomyomas (1, 2).

Observed mainly in women of childbearing age (3), LAM can be associated with tuberous sclerosis complex (TSC-LAM) involving germline mutations of the *TSC1* and *TSC2* genes, or in sporadic form involving somatic mutations of the *TSC2* gene (2). Mutations of *TSC1* or *TSC2* – both involved in the regulation of cell growth - lead to the constitutive activation of the mTOR signaling pathway, which causes the uncontrolled multiplication of LAM cells. Shortness of breath, pneumothorax and decreased exercise capacity are usually the first symptoms of LAM (2).

The prevalence of LAM is estimated at 3.4 to 7.8 per million women worldwide, and its incidence reaches 0.23 to 0.31 per million women per year (4). In 2015 in France, an estimated 200 to 250 patients were affected, according to the French registry “ReLAMce,” including 30% of TSC-LAM (5). The impact of LAM on patients’ quality of life can be significant, with prognosis being determined by the rapidity of decline in respiratory function (6). In a recent study of 574 patients, the 5-year and 8-year cumulative survival rates from the onset of symptoms were 97.6 and 87.1%, respectively (7). Lung transplantation is a possible outcome– although increasingly rare since the introduction of sirolimus (also called *rapamycin*) (8–11) –, usually before the age of 65 years (12).

Diagnosis is made by a combination of clinical signs and characteristic high-resolution computed tomography, less commonly by tissue biopsy (usually lung biopsy, lymph node or lymphangioleiomyoma biopsy). Vascular endothelial growth factor (VEGF)-D level – when ≥800 pg./mL – confirms the diagnosis of LAM in the presence of a suggestive radio-clinic presentation (13).

LAM management used to be symptomatic and focused on the clinical consequences of the disease. Advances in molecular understanding have paved the way for therapeutic strategies with agents such as sirolimus, an inhibitor of the mTOR pathway. The landmark Multicenter International LAM Efficacy of Sirolimus (MILES) Trial in 2008 demonstrated that sirolimus stabilized lung function, reduced serum VEGF-D, improved functional performance and was associated with a reduction in symptoms and improvement in quality of life compared with placebo over 1 year; the benefits waned when the drug was withheld in the second year (14). In 2016, the American Thoracic Society (ATS) and Japanese Respiratory Society (JRS) released joint clinical practice guidelines which were endorsed by the LAM Foundation (15) and later by the French recommendations (16). Key recommendations included the following: for patients with LAM and abnormal/declining lung function, treatment with sirolimus rather than observation is recommended; for selected patients with LAM and problematic chylous effusions, treatment with sirolimus before invasive management is recommended.

To describe the epidemiological and outcomes of patients with LAM treated with sirolimus in France, this observational and retrospective study was performed using the National healthcare database (SNDS, *Système National des Données de Santé*). This study was conducted in response to a request from the French health authorities, namely the *Haute Autorité de Santé*, to answer several questions related to the use of sirolimus in patients with LAM in real-life setting^1^.

## Methodology

### Data sources

The SNDS collates pseudonymized health data collected by public bodies in France.The SNDS contains individual-level data for outpatient and private healthcare facilities health expenditure billing and reimbursement purposes, linked to the hospitalization database (PMSI) by a unique, pseudonymous identifier. This means pseudonymized individual-level data for all healthcare claims for over 99% of the population residing in France, whatever their insurance scheme, i.e., almost 65 million people (17–20).

### Study population

Since LAM does not benefit from a specific ICD-10 code, patients were identified through a stepwise algorithm considering sirolimus indications and uses in real-life as well as the occurrence of events associated with the evolution of the disease. Thus, adult (≥18Y) women with a unique identifier and at least one dispensing of sirolimus between January 1st, 2014, and December 31st, 2021, in the SNDS were included in the study population. All patients with at least one ICD-10 code related to another indication down to 8 years prior to first sirolimus identification and up to 2 months after were excluded (see Supplementary Table S3). This population is referred to as patients with “possible” LAM within the manuscript.

In order to have a more specific identification of patients with LAM in France, a subgroup was built integrating patients with at least one clinical manifestation highly suggestive of LAM (including pneumothorax, ascites, respiratory failure) in the 8 years prior to the index date and up to 1 year after. Clinical events and associated ICD-10, DRG (Diagnosis Related Group), and CCAM (*Classification Commune des Actes Médicaux*) codes associated are listed in Supplementary Table S1 (such as pneumothorax, ascites, respiratory failure). This subgroup is referred to as patients with “probable” LAM. Among those, all patients with a diagnosis of TSC-LAM, defined by the presence of at least one hospitalization with the ICD-10 code Q851 Tuberous Sclerosis Complex at any time during the whole inclusion period, were identified.

### Study period

Patients were included between January 1^st^, 2014, and December 31^st^, 2021. The index date was the first delivery of sirolimus during the study period. Each patient was followed-up until kidney or lung transplantation, until death, absence of healthcare consumption for 3 continuous years, or the end of the study period (December 31^st^, 2021), whichever came first. An 8-year follow-back period was considered to assess medical history.

### Outcomes

Patients’ characteristics were described at the index date and comorbidities captured from January 1st, 2006. Diagnosis to treatment time was established as the delay between the first event confirming LAM or TSC-LAM, and the first dispensing of sirolimus.

The number of prevalent and incident LAM patients was estimated by calendar year over the follow-up period. The yearly incidence and prevalence rates per 1,000,000 French adult women were estimated considering INSEE data.

Evolution of LAM was evaluated through lung or kidney transplantation, death from all causes, pneumothorax, respiratory failure, pleural complications, ascites, and hospitalization for pleural intervention, angiomyolipoma, hemangioma, hemoperitoneum, meningioma, lymphangioma, lymphatic complications, or emphysema (see Supplementary Table S2).

Safety profile was assessed through the occurrence of opportunistic infections, pneumonia, lymphoma or skin cancer, drug-induced interstitial lung disease, multifocal leukoencephalopathy, cytopenia, hypercholesterolemia, hypertensive flare-ups, and respiratory tract hemorrhage.

### Statistical methods

Continuous quantitative variables were summarized using mean ± standard deviation (SD), median, 1^st^ and 3rd quartiles [Q1; Q3]. Categorical variables were presented as numbers and percentages.

Survival estimates were calculated using the Kaplan–Meier method based on the entire study population for the assessment of adverse events and disease complications events with the exception of death. Only patients affiliated to the general scheme were used as death could be less reliably collected within the other insurance schemes during the first years of the study period. A number of associated summary statistics including median survival time and in particular survival rates every 6 months from 0 to 8 years were estimated with corresponding 2-sided 95% CIs. When reached, confidence intervals for median survival time were computed with the Brookmeyer and Crowley method and the CIs for the survival function estimates at the time points defined above were derived using the log–log transformation according to Kalbfleisch and Prentice with back transformation to a CI on the untransformed scale. The estimate of the standard error was computed using Greenwood’s formula. The 10% Pocock rule was applied to curtail the survival curves. Time to event was defined as the time from the index date to the occurrence of the first adverse event or complication and was censored at the end of follow-up. Restricted mean survival time with a time horizon defined by the last know event was also provided to summarize the survival profile.

Statistical analyses were performed with SAS® (version 9.4).

## Results

### Patient selection

**Figure 1:**
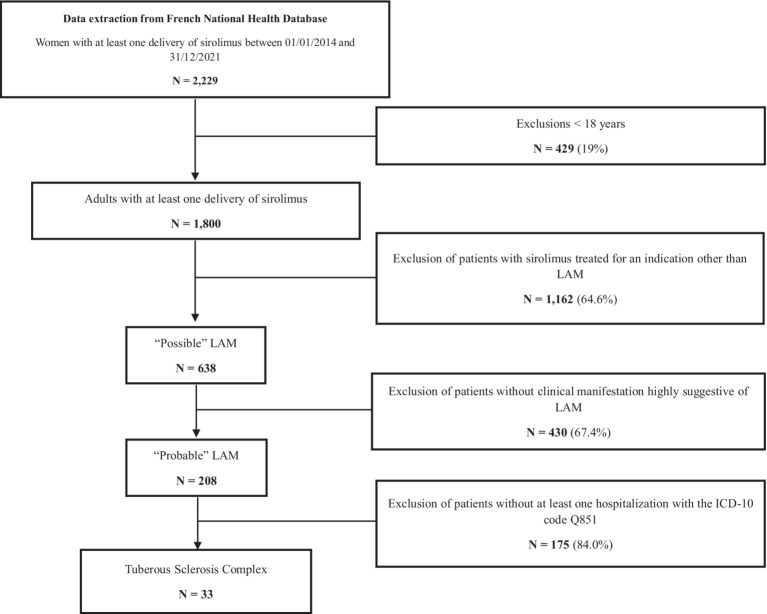
Flowchart of patients’ selection.

The flow-chart of the study is presented in Figure 1. Over the entire study period, 638 patients were considered as treated with sirolimus for possible LAM, including 208 patients treated for “probable” LAM and 33 patients for TSC-LAM (Figure 1). Exclusion criteria met by patients are provided in Supplementary Table S3 (one patient could meet several criteria). The intersection plot in Supplementary Figure S1 displays combinations of LAM clinical manifestations found in the probable LAM cohort.

### Prevalence and incidence

**Figure 2:**
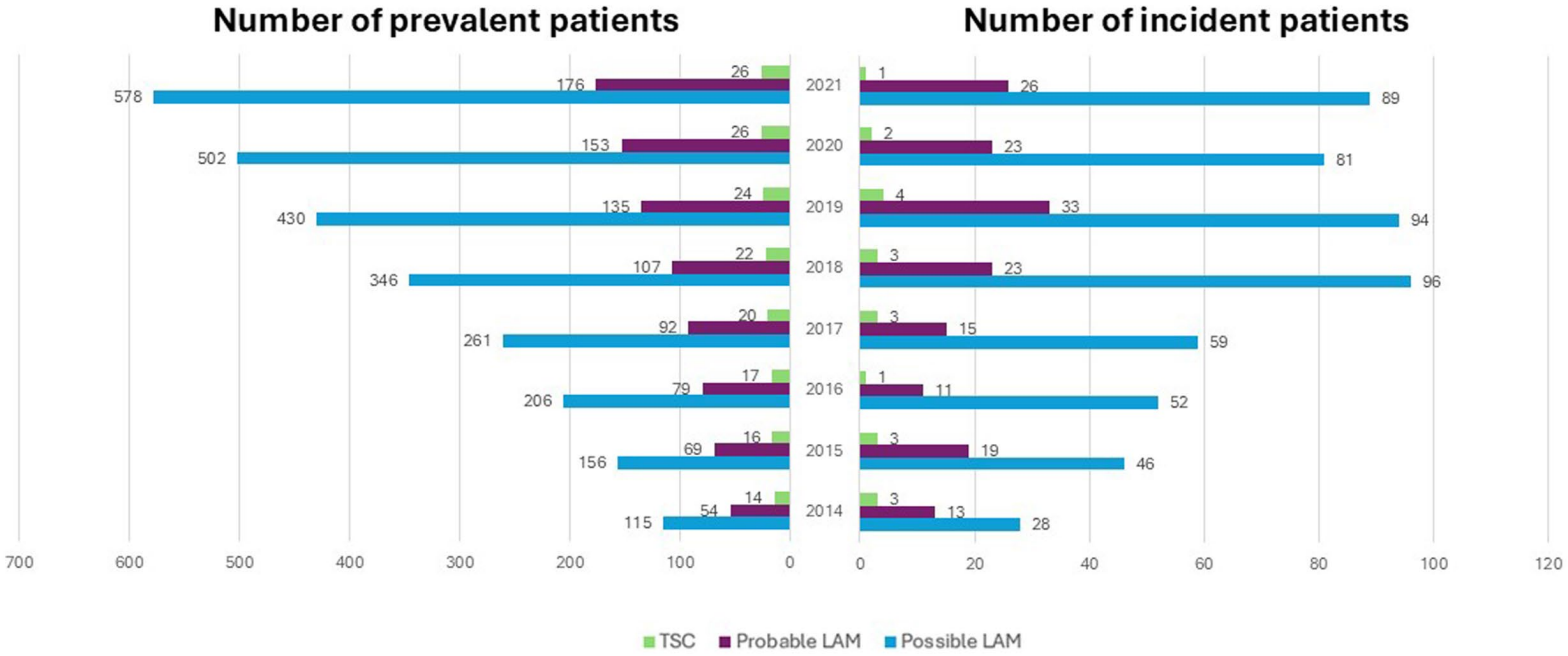
Number of prevalent and incident LAM patients treated with sirolimus in France between 2014.

In 2021, 89 incident patients with possible LAM were identified, including 26 patients with probable LAM and 1 patient with TSC-LAM (Figure 2). Overall, the number of incident patients varied from 28 to 96 each year for possible LAM, from 11 to 33 each year for probable LAM and from 1 to 4 patients each year for TSC-LAM patients. In 2021, the incidence rate of patients treated with sirolimus for probable LAM in France was estimated at 0.9 per 1,000,000 French adult women.

In 2021, there were 578 possible LAM prevalent patients in France, amongst whom 176 were considered probable LAM, and 26 TSC-LAM. The prevalence rate of patients treated with sirolimus for probable LAM in France was estimated at 6.3 per 1,000,000 French adult women Supplementary Table S4.

### Patients’ characteristics

Median [Q1; Q3] age at index date was 45.0 years [34.0; 58.5] for patients with probable LAM and 40.0 years [28.0; 56.0] for patients with TSC-LAM (see Supplementary Table S5). Most patients were aged between 35 and 44 years among the probable LAM patients (*n* = 44, 21%) and patients with TSC-LAM (*n* = 11, 33%), whereas possible LAM patients were older, with most patients within the 65+ group (*n* = 135, 21%).

The most commonly observed comorbidities identified using label of ICD-10 codes were systemic hypertension (*n* = 60 (29%) patients with probable LAM, and *n* = 10 (30%) patients with TSC-LAM), treatment for vascular risk (*n* = 42 (20%) probable LAM and *n* = 7 (21%) patients with TSC-LAM), malnutrition, which exceeded 22% in the probable LAM cohort (*n* = 47).

The mean (±SD) duration of treatment with sirolimus was: 2.9 years (±2.81) for probable LAM and 3.7 years (±2.73) for TSC-LAM patients. Twenty-one patients were also exposed to everolimus over the study follow-up period, including 19 probable LAM and 9 TSC-LAM patients. Mean (±SD) follow-up duration was 3.67 years (±2.81) for probable LAM and 4.58 (±2.65) for TSC-LAM patients.

As a proxy of the time between diagnosis and treatment with sirolimus, the mean (±SD) time between the first criteria defining the probable LAM / TSC-LAM and the index date was estimated on average at about 3.8 years (±2.8) for probable LAM and 4.8 years (±2.8) for TSC-LAM patients.

Seventeen (13.0%) deaths occurred in the probable LAM group and 6 (18.2%) in the TSC-LAM group (including here deaths also occurring after lung transplantation). Sixteen patients (7.7%) were transplanted in the probable LAM group and 2 (6.1%) in the TSC-LAM group.

### Disease evolution

Table 1 describes the outcomes of patients presenting at least one event of disease worsening or death during the follow-up period. As the study data date from before 2014, mean survival is calculated on general scheme affiliated patients only, to ensure that all deaths go backwards. Thus, in the survival population, there are 171 patients instead of 208, and 23 deaths are recorded instead of 27.

**Table 1:** LAM disease evolution outcomes.

Variable	Restricted mean survival time (SE)	Probable LAM	TSC-LAM
**N**		208	33
Death (all causes)	6.80 (+/− 0.20)	27 (13.0%)	6 (18.2%)
Reason about hospitalisation			
Respiratory failure	3.66 (+/− 0.09)	28 (13.5%)	2 (6.1%)
Lung transplant	7.02 (+/− 0.15)	16 (7.7%)	2 (6.1%)
Lymphatic complications	4.66 (+/− 0.09)	15 (7.2%)	0
Pneumothorax or pleural drainage	5.32 (+/− 0.08)	9 (4.3%)	1 (3.0%)
Lymphangioleioma	0.71 (+/− 0.01)	8 (3.9%)	0
Pleural draining / pleural surgery	3.65 (+/− 0.04)	6 (2.9%)	0
Pleural complication	1.49 (+/− 0.01)	5 (2.4%)	0
Pneumothorax	5.43 (+/− 0.06)	5 (2.4%)	1 (3.0%)
Ascite	0.71 (+/− 0.00)	3 (1.4%)	0
Emphysema	0	0	0
Hemoperitoneum	0	0	0
Renal transplant	0	0	0

The 5-year survival after sirolimus initiation (index date) was 84% (95% CI: 76%; 90%) for probable LAM patients, and 77% (95% CI: 48%; 91%) for TSC-LAM patients. The 2-year survival after 1st manifestation of LAM was 91% (CI: 84%;95%) for probable LAM patients.

**Figure 3:**
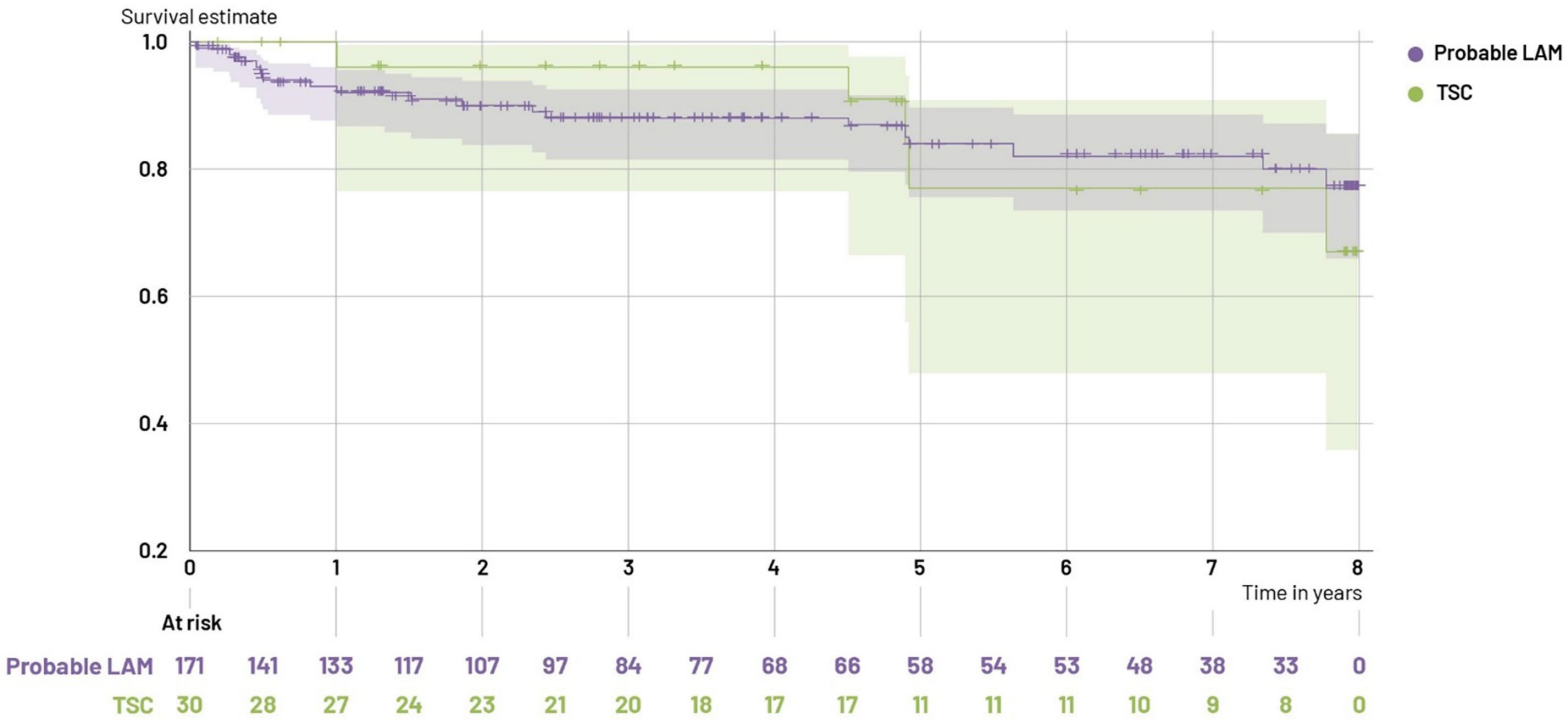
Kaplan Meier estimates of overall survival.

Furthermore, for probable LAM patients, the 5-year survival after the index date was 93% (CI 82%; 97%) for the subgroup of patients aged between 18 and 50 years, and 70% (55%; 81%) for the subgroup of patients aged 51 years and over (Figure 3).

Hospitalizations for respiratory failure: the probability to remain free of hospitalization for respiratory failure 5 years after sirolimus initiation was 81% (74–87%) for probable LAM patients. A total of 89 hospitalizations for respiratory failure were identified during the follow-up for 30 patients.

Hospitalizations for lymphatic complications: the probability to remain free of hospitalization for lymphatic complications 5 years after sirolimus initiation was 90% (CI: 83–94%) for probable LAM. A total of 39 hospitalizations for lymphatic complications were identified during the follow-up for 17 patients. Hospitalizations for lymphatic complications occurred mainly during the second year of follow-up for probable LAM.

Lung transplant: at 5-years after initiation of sirolimus, the probability of undergoing lung transplant was 8% (CI: 5–14%) in probable LAM (see Supplementary Figure S2) and 6% (CI: 2–22%) in patients with TSC-LAM. Among the 16 patients with probable LAM who underwent lung transplantations during the follow-up period, most of them (12/16, 71%) occurred within the first 2-years after sirolimus initiation.

### Safety profile

Table 2 presents the occurrence of pre-selected severe adverse events identified in the SNDS during follow-up.

**Table 2:** Occurrence of severe adverse events of interest.

Severe adverse events of interest	Probable-LAM	TSC-LAM
**N**	208	33
Cytopenia requiring hospitalization:	10 (4.8%)	2 (6.1%)
Anemia	8 (3.9%)	1 (3.0%)
Neutropenia	2 (1.0%)	1 (3.0%)
Thrombocytopenia	0	0
Pneumonia requiring hospitalizations	16 (7.7%)	1 (3.0%)
Opportunistic infections requiring hospitalization	10 (4.8%)	2 (6.1%)
Initiation of lipid lowering therapies within 6 M after sirolimus initiation	7 (3.4%)	2 (6.1%)
Lymphomas or skin cancers	3 (1.4%)	0
Respiratory tract hemorrhage requiring hospitalization	1 (0.5%)	0
High blood pressure flare-ups requiring hospitalization	1 (0.5%)	1 (3.0%)
Hospitalization for hypercholesterolemia after sirolimus initiation	1 (0.5%)	0
Hospitalization for hypercholesterolemia within 1 year after sirolimus initiation	1 (0.5%)	0
Multifocal leukoencephalopathies requiring hospitalization	0	0
Drug-induced interstitial lung disorders	0	0

A total of 12 probable LAM patients (including 2 TSC-LAM patients) experienced cytopenia during the follow-up period, most of them being anemia (*n* = 8). In total, 17 patients had pneumonia requiring hospitalization, including 16 probable LAM and 1 patient with TSC-LAM. Among the probable LAM patients, 7 patients initiated lipid lowering therapy within 6 months after the index date and 2 among the TSC-LAM subgroup.

## Discussion

This study enabled us to gain knowledge related to LAM and its treatment with sirolimus. Data collected and analyzed included epidemiological data, patients’ characteristics amongst which comorbidities, evolution of the disease and worsening events data. In 2021 according to this study, there were 578 possible LAM cases in France, of which 176 were considered probable LAM, and 26 TSC-LAM. The order of magnitude of these figures is consistent with estimates from the French literature, considering that the present study only refers to treated patients (5). While increased prevalence was evidenced for possible and probable LAM, progression was much slower for TSC-LAM patients, probably due to the rarity of the disease, and the more frequent deaths in this group.

Sirolimus is indicated as a first-line treatment in sporadic LAM with forced expiratory volume in one second (FEV1) < 70% of predicted value, or with a yearly decline in FEV1 greater than 90 mL/year (16). In our study, patients treated with sirolimus were young (median age 45 years) but 13% of sirolimus-treated patients died during the follow-up period in the probable LAM group; deaths were more frequent in TSC-LAM patients (18.2%). Of note, results from a 16-year Italian observational study including 162 patients provided evidence supporting the long-term efficacy of sirolimus (9). One should notice that all-cause deaths were collected to report mortality in this study: we could not determine whether the patients died of a cause directly related to LAM or due to another reason, as we did not have access to the precise cause of death. The relatively high rate of death and lung transplantation in this study may be related to selection bias, i.e., the patients requiring treatment, and being hospitalized at least once in the disease course.

The most frequent non-fatality events of interest were related to the respiratory system. Lung transplant at 5-years after initiation of sirolimus did not reproduce the greater severity expected for TSC-LAM patients, since the probability of having a lung transplant was 8% in probable LAM patients compared to only 6% in patients with TSC-LAM. Small numbers and early mortality in the TSC-LAM group may account for these results with other confounding factors such as contra-indication for lung transplant in TSC-LAM patients. One striking observation was that the number of worsening events was of the same order of magnitude as the number of deaths for TSC-LAM but not for probable patients. Ninety-five worsening events and 27 deaths were reported for the probable LAM group; the ratio was equal to 1 for the TSC-LAM group, since there were 6 worsening events for 6 deaths. We might have expected a greater number of hospitalizations and worsening events in the months or years preceding death. Of note is the absence of lung transplantation - counted as a disease worsening event - in TSC patients; patients with TSC-LAM may often be denied lung transplantation for neurological and/or severe comorbidities.

Time between LAM diagnosis and initiation of treatment remained quite long, partially explained by the diagnostic wandering frequently associated with patients’ pathways in a context of rare diseases (21). A duration of 3–4 years is consistent with some previous series, with an interval of 2 years between the onset of symptoms and diagnosis for a 1998–2001 period (1), and a median time of 4.3 years (ranging from a few days to 15 years) for an early 1990s series of cases (22). In a proportion of the patients, indication is based on observed decline in FEV1, which warrants additional observation time before treatment can be initiated.

This study has several strengths. First, a study based on the SNDS is not limited to patients registered in a registry: the SNDS covers the entire French population (17). The primary objective of the study was descriptive; therefore, no power or sample size calculations were required. Secondly, as this study is based on a secondary use of data collected mainly for medico-economic purposes, there was no information bias on the part of patient or physician reporting: the database is supposed to display reality of treatment dispensations, through reimbursement data. Third, the depth of the historical data, as well as the chaining of reimbursed outpatient care to hospital data are particularly useful for this kind of project.

Some limitations can be underlined, mainly measurement bias. First, only treatments and procedures covered by the national health insurance were recorded. Over-the-counter drugs or drugs prescribed but not reimbursed, as well as procedures not reimbursed were not considered in the study. Besides, only drug dispensation data could be accessed. Therefore, we could only assume that patients effectively took the drug, and that they did so on the day of dispensation. Additionally, the database does not contain medical results such as pulmonary function tests, laboratory tests, imaging procedures or even specific coding for LAM. For this reason, we have been using algorithms to identify patients with sporadic-LAM and TSC-LAM and their outcomes. The origin of complications (such as for lymphatic complications for instance) cannot be assessed within this database and we might thus over-estimate these complications in our study as they could be linked to the LAM disease itself, or to other reasons (hardly differentiable in the SNDS). To be noted also that only aggregated data at the healthcare facility level could be obtained for VEGF-D testing so this could of be used as part of the algorithm (no data at the patient’s level). Within the PMSI database, data are collected and entered for reimbursement purposes mainly, and code assignment can be influenced by reimbursement policy. This can introduce information bias if doctors transmit only the information necessary for billing, which may adequately describe the medical effort carried out during the hospital stay and omit other relevant medical data. Only severe forms of comorbidities and medical events (such as LAM disease worsening events or pre-selected adverse events identifiable within the SNDS in our study) could be identified through hospital stays for each comorbidity/event. This may have underestimated the presence of other comorbidities and events in our study population, particularly the least severe events. Similarly, the least severe side effects of sirolimus (such as mucositis, edema, diarrhea and menstrual disturbances) were likely underreported, as they would rarely affect the billing of hospital stays. This is also why this study focused on severe adverse events. Finally a selection bias could not be excluded. Thus, as in the SNDS, the classification used is the ICD-10 (and not ICD-10-CM) with only an unspecific 3-digit code J848 (Other specified interstitial pulmonary diseases) an algorithm was developed to identify patients. This algorithm was built with an expert committee and took into account possible and probable LAM. Regarding data from other datasources in France, on May 1st, 2023, 439 patients with LAM (treated or not) were registered in the national rare diseases database (BNDMR, *Banque Nationale de Données Maladies Rares*), a system distinct from the SNDS that only comprises cases seen in rare disease centers (reference/competence centers)^2^. Even though the different time referential and methods used between our study and the BNDMR does not allow direct comparison, our results are of the same order of magnitude. However, as the BNDMR also includes patients receiving everolimus (prescribed for neurological or renal purpose) alone and non-treated patients, it is conceivable that the prevalent number of possible LAM patients – restricted to the ones treated with sirolimus only – retrieved in the present study may have been overestimated, and that our algorithms may not have been restrictive enough.

In addition, the history of information and insurance schemes in the SNDS bases have evolved over time; from 2006 and for a few years, only the data of individuals affiliated to the general scheme was reported in the health insurance databases (as death could be less reliably collected within the other insurance schemes during the first years of the study period)^3^ (18). Therefore, as LAM is a slowly progressive disease, it is possible that patients on other regimens may not have been captured. Finally, we may also have underestimated patients with confirmed LAM if the event leading to diagnosis occurred more than 8 years prior to the index date.

## Conclusion

This study provides an updated epidemiological estimate of LAM patients treated with sirolimus in France between 2014 and 2021. Even though some of the results should be interpreted cautiously in the light of limitations related to the use of claims database, evolution of the disease and missing safety data, the information retrieved in this study is very valuable, as few studies provide real-world information on LAM populations. Among 208 patients treated with sirolimus for an estimated probable, sporadic LAM, or TSC-LAM, the 5-year survival after sirolimus initiation was 84, 13.5% of patients had respiratory failure and 7.7% had undergone lung transplantation. Serious adverse effects were infrequent. Studies covering longer periods should be conducted to consider long-term efficacy data, survival in particular, and safety data. But also, comparative studies must be carried out to assess the long-term effectiveness of sirolimus. We recommend additional research on SNDS data, which in the future could be linked to a diagnosis of LAM in the individual patients owing to the BNDMR database. Collecting real-life data on this disease contributes to delineate its evolution, treatment modalities, and outcome.

## Data Availability

The data analyzed in this study is subject to the following licenses/restrictions: The data are not publicly available and were used under license for the current study. Requests to access these datasets should be directed to https://www.assurance-maladie.ameli.fr/etudes-et-donnees/en-savoir-plus-snds/utilisation-accompagnement-donnees-snds.

## Footnotes

^1^CT-17471_RAPAMUNE_PIC_EI_Avis2_CT17471.pdf [Internet]. [cité 22 mai 2024]. Available at: https://www.has-sante.fr/upload/docs/evamed/CT-17471_RAPAMUNE_PIC_EI_Avis2_CT17471.pdf.

^2^Nombre de cas par maladie rare dans la BNDMR | Banque Nationale de Données Maladies Rares [Internet]. [cité 13 mai 2024]. Available at: https://www.bndmr.fr/publications/nombre-de-cas-par-mr/.

^3^Historique des données du SNDS | Documentation du SNDS & SNDS OMOP [Internet]. [cité 16 mai 2024]. Available at: https://documentation-snds.health-data-hub.fr/snds/fiches/historique_donnees.html#historique-de-l-information-et-des-regimes-dans-les-bases-du-snds.
