# Supplementary material for: Characteristics and outcomes of patients with LAM receiving sirolimus in France based on real-life data
Source: Front Med (Lausanne). 2025 Jan 8;11:1494713. doi: 10.3389/fmed.2024.1494713 (PMC11753350; doi:10.3389/fmed.2024.1494713)
Supplement: Supplementary file 1 [file Data_Sheet_1.pdf]

Supplementary Table S1 - Clinical manifestations in the 8 years prior to the index date and up to 1 year after, which identifies Probable LAM patients

**Clinical manifestations**	**Operational definitions**
Tuberous Sclerosis Complex	Presence of ICD10 code : Q851 (Tuberous sclerosis as DP/DR/DAS)
Pneumothorax	Patients with at least one hospitalization stay with one of the following ICD-10 or CCAM codes<br>J930 Spontaneous tension pneumothorax<br>J931 Other spontaneous pneumothorax<br>J938 Other pneumothorax<br>J939 Pneumothorax, unspecified<br>04M12 Pneumothorax
Ascites	At least one hospital stay in MCO with ICD10 codes (DP/DR/DAS):<br>R18 : Ascites
Respiratory failure	Patients with at least one hospitalization stay with one of the following ICD10 codes:<br>J80 Adult respiratory distress syndrome<br>J81 Pulmonary oedema<br>J84 Other interstitial pulmonary diseases
Pleural draining/ pleural surgeries	Patients with at least one hospitalization stay with one of the CCAM codes for pleural draining/pleural surgeries:<br>GGJB002 Evacuation of a pleural effusion, transcutaneous without guidance<br>GGJB001 Drainage of pleural effusion, transcutaneous without guidance<br>GGLB006 Placement of a thoracic drain for pleural lavage, transcutaneous approach<br>GGJB005 Placement of several thoracic drains for pleural irrigation-washing, transcutaneously<br>GGJB007 Pleural lavage session, using an implanted device<br>GGJB006 Pleural irrigation-washing session<br>GGJA002 Evacuation of collection from pleural cavity, by thoracotomy<br>GGJA004 Collection evacuation of pleural cavity with costal resection, by thoracotomy<br>GGJC001 Evacuation of septic collection from pleural cavity with debridement, by thoracoscopy<br>GGJA001 Evacuation of septic collection from pleural cavity with debridement, by thoracotomy<br>GGJC002 Evacuation of hemothorax, by thoracoscopy<br>GGJA003 Debridement of pleural cavity, by thoracotomy
Lymphatic complications	Patients with at least one hospitalization stay with one of the ICD-10 or CCAM codes for lymphatic complications:<br>I89 : Other non-infectious disorders of lymphatic vessels and lymph nodes
Lung transplant	Patients with at least one hospitalization stay with one of the ICD-10 (DP/DR) or CCAM or GHM codes during the follow-up

Clinical manifestations	Operational definitions
	ICD10 : Z942 : Lung transplantation<br>CCAM : GFEA001 : SEQUENTIAL TRANSPLANTATION OF 2 LUNGS, BY THORACOTOMY WITH CEC<br>CCAM : GFEA002 : LUNG LOBE TRANSPLANT, BY THORACOTOMY WITH CEC<br>CCAM : GFEA003 : LUNG TRANSPLANTATION, BY THORACOTOMY WITHOUT CEC<br>CCAM : GFEA004 : SEQUENTIAL TRANSPLANTATION OF 2 LUNGS, BY THORACOTOMY WITHOUT CEC<br>CCAM : GFEA005 : LUNG LOBE TRANSPLANTATION, BY THORACOTOMY WITHOUT CEC<br>CCAM : GFEA006 : BIPULMONARY TRANSPLANTATION, BY THORACOTOMY WITH CEC<br>CCAM : GFEA007 : LUNG TRANSPLANTATION, BY THORACOTOMY WITH CEC<br>GHM : 04M21 : Lung transplant follow-ups
Other disorder of lung	Presence of ICD10 code: J984
Angiomyolipoma	At least one hospital stay in MCO with ICD10 codes (DP/DR):<br>D177 : Benign lipomatous tumor of other sites<br>D179 : Benign lipomatous tumor, unspecified<br>D171 : Benign lipomatous tumor of the skin and subcutaneous tissue of the trunk
Meningioma	At least one hospital stay in MCO with ICD10 codes (DP/DR):<br>D32: Benign meningeal tumor
Hemoperitonea	At least one hospital stay in MCO with ICD10 codes (DP/DR):<br>K661 : Hemoperitoneum
Lymphangioma	At least one hospital stay in MCO with ICD10 codes (DP/DR):<br>D181 Lymphangioma

Precision : in the SNDS, the classification used is the ICD-10 (and not ICD-10-CM).

Supplementary Table S2 - Description of variables – primary and secondary outcomes

VARIABLE	ROLE	DATA source(s)	OPERATIONAL DEFINITION
Lung transplant	Main outcome	SNDS	Patients with at least one hospitalization stay with one of the ICD-10 (DP/DR) or CCAM or GHM codes during the follow-up<br><br>ICD10 : Z942 : Lung transplantation<br>CCAM : GFEA001 : SEQUENTIAL TRANSPLANTATION OF 2 LUNGS, BY THORACOTOMY WITH CEC<br>CCAM : GFEA002 : LUNG LOBE TRANSPLANT, BY THORACOTOMY WITH CEC<br>CCAM : GFEA003 : LUNG TRANSPLANTATION, BY THORACOTOMY WITHOUT CEC<br>CCAM : GFEA004 : SEQUENTIAL TRANSPLANTATION OF 2 LUNGS, BY THORACOTOMY WITHOUT CEC<br>CCAM : GFEA005 : LUNG LOBE TRANSPLANTATION, BY THORACOTOMY WITHOUT CEC<br>CCAM : GFEA006 : BIPULMONARY TRANSPLANTATION, BY THORACOTOMY WITH CEC<br>CCAM : GFEA007 : LUNG TRANSPLANTATION, BY THORACOTOMY WITH CEC<br>GHM : 04M21 : Lung transplant follow-ups
Renal transplant	Main outcome	SNDS	Patients with at least one hospitalization stay with one of the following GHM or CCAM codes<br>GHM : 27C06 Kidney transplantation<br>CCAM : JAEA003 Kidney transplantation<br>CCAM : HNEA002 Pancreas and kidney transplant, by laparotomy
Death (all causes)	Main outcome	SNDS	All deaths (intra and out of hospital deaths) of any causes
Pneumothorax	Main outcome	SNDS	Patients with at least one hospitalization stay with one of the following ICD-10 or CCAM codes<br>J930 Spontaneous tension pneumothorax<br>J931 Other spontaneous pneumothorax

VARIABLE	ROLE	DATA source(s)	OPERATIONAL DEFINITION
			J938 Other pneumothorax<br>J939 Pneumothorax, unspecified<br>04M12 Pneumothorax
Hospitalization for pleural draining / pleural surgeries	Main outcome	SNDS	Patients with at least one hospitalization stay with one of the CCAM codes for pleural draining/pleural surgeries:<br>GGJB002 Transcutaneous evacuation of pleural effusion without guidance<br>GGJB001 Drainage of pleural effusion, transcutaneous without guidance<br>GGLB006 Placement of a thoracic drain for pleural lavage, transcutaneously<br>GGJB005 Placement of several thoracic drains for pleural irrigation-washing, transcutaneously<br>GGJB007 Pleural lavage session, using an implanted device<br>GGJB006 Pleural irrigation-washing session<br>GGJA002 Evacuation of collection from pleural cavity, by thoracotomy<br>GGJA004 Collection evacuation of pleural cavity with costal resection, by thoracotomy<br>GGJC001 Removal of septic collection from pleural cavity with debridement, by thoracoscopy<br>GGJA001 Evacuation of septic collection from pleural cavity with debridement, by thoracotomy<br>GGJC002 Evacuation of hemothorax, by thoracoscopy<br>GGJA003 Debridement of pleural cavity, by thoracotomy
Respiratory failure	Main outcome	SNDS	Patients with at least one hospitalization stay with one of the following ICD10 codes:<br>J80 Adult respiratory distress syndrome<br>J81 Pulmonary oedema<br>J84 Other interstitial pulmonary diseases
Pleural complications	Main outcome	SNDS	At least one hospital stay in MCO with ICD10 codes (DP/DR):<br>J90: Pleural effusion, not elsewhere classified”.

VARIABLE	ROLE	DATA source(s)	OPERATIONAL DEFINITION
			J94: Other pleural disorders<br>R091: Inflammation of pleura
Ascites	Main outcome	SNDS	At least one hospital stay in MCO with ICD10 codes (DP/DR):<br>R18 : Ascites
Hospitalization for angiomyolipoma	Main outcome	SNDS	At least one hospital stay in MCO with ICD10 codes (DP/DR):<br>D177 : Benign lipomatous tumor of other sites<br>D179 : Benign lipomatous tumor, unspecified<br>D171 : Benign lipomatous tumor of the skin and subcutaneous tissue of the trunk
Hospitalization for hemangioma	Main outcome	SNDS	At least one hospital stay in MCO with ICD10 codes (DP/DR):<br>D180 : Hemangioma, any site
Hospitalization for hemoperitonea	Main outcome	SNDS	At least one hospital stay in MCO with ICD10 codes (DP/DR):<br>K661 : Hemoperitoneum
Hospitalization for meningioma	Main outcome	SNDS	At least one hospital stay in MCO with ICD10 codes (DP/DR):<br>D32 : Benign meningeal tumor
Hospitalization for lymphangioma	Main outcome	SNDS	At least one hospital stay in MCO with ICD10 codes (DP/DR):<br>D181 Lymphangioma
Hospitalization for lymphatic complications	Main outcome	SNDS	Patients with at least one hospitalization stay with one of the ICD-10 or CCAM codes for lymphatic complications:<br>I89 : Other non-infectious diseases of vessels and lymph nodes
Hospitalization for Emphysema	Main outcome	SNDS	Patients with at least one hospitalization stay with one of the ICD-10 or CCAM codes for lymphatic complications<br>J982 Pseudocystic Emphysema<br>J43 Pseudocystic Emphysema
Prevalent patients: Number of LAM patients treated with sirolimus between January 2014 and December 2021	Secondary outcome – Number of LAM patients treated with sirolimus	SNDS	All patients with at least one delivery of sirolimus and meeting the inclusion / exclusion criteria between January 2014 and December 2021, including ROM/COM
Incident patients:	Secondary outcome –	SNDS	All patients with at least one delivery of sirolimus (and no delivery during the

VARIABLE	ROLE	DATA source(s)	OPERATIONAL DEFINITION
Number of LAM patients newly treated with sirolimus between January 2014 and December 2021	Number of LAM patients treated with sirolimus		previous years) and meeting the inclusion / exclusion criteria between January 2014 and December 2021, including ROM/COM
Opportunistic infections requiring hospitalization	Secondary outcome	SNDS	All patients date with at least one hospital stay with one of the following ICD10 codes after index date (DP/DR):<br>B25 Cytomegaloviral disease<br>B27.1 Cytomegaloviral mononucleosis<br>B00 Herpesviral [herpes simplex] infections<br>B01 Varicella [chickenpox]<br>B02 Zoster [herpes zoster]<br>A60.0 Herpesviral infection of genitalia and urogenital tract<br>B27.0 Gammaherpesviral mononucleosis<br>A81.2 Progressive multifocal leukoencephalopathy<br>B17.9 Acute viral hepatitis, unspecified<br>G02.0 Meningitis in viral diseases classified elsewhere<br>J17.1 Pneumonia in viral diseases classified elsewhere<br>A16 Respiratory tuberculosis, not confirmed bacteriologically or histologically<br>A17 Tuberculosis of nervous system<br>A18 Tuberculosis of other organs<br>A19 Miliary tuberculosis<br>A31 Infection due to other mycobacteria<br>K23.0 Tuberculous oesophagitis<br>K67.3 Tuberculous peritonitis<br>K93.0 Tuberculous disorders of intestines, peritoneum and mesenteric glands<br>M01.1 Tuberculous arthritis<br>M90.0 Tuberculosis of bone<br>N33.0 Tuberculous cystitis<br>N74.0 Tuberculous infection of cervix uteri<br>N74.1 Female tuberculous pelvic inflammatory disease<br>A44 Bartonellosis

VARIABLE	ROLE	DATA source(s)	OPERATIONAL DEFINITION
			A48.1 Legionnaires disease<br>A48.2 Nonpneumonic Legionnaires disease [Pontiac fever]<br>A40.3 Sepsis due to Streptococcus pneumoniae<br>J13 Pneumonia due to Streptococcus pneumoniae<br>B95.3 Streptococcus pneumoniae as the cause of diseases classified to other chapters<br>A43 Nocardiosis<br>A42 Actinomycosis<br>A32 Listeriosis<br>A02 Other salmonella infections<br>B37 Candidiasis<br>B39 Histoplasmosis<br>B40 Blastomycosis<br>B44 Aspergillosis<br>B45 Cryptococcosis<br>B48.5 Pneumocystosis<br>G02.1 Meningitis in mycoses<br>J17.2 Pneumonia in mycoses<br>A07.2 Cryptosporidiosis<br>A07.3 Isosporiasis<br>B55 Leishmaniasis<br>B58 Toxoplasmosis<br>B78 Strongyloidiasis
Pneumonia	Secondary outcome	SNDS	All patients date with at least one hospital stay with one of the following ICD10 codes after index date (DP/DR):<br>J13 Pneumonia due to Streptococcus pneumoniae<br>J150 Pneumonia due to Klebsiella pneumoniae<br>J151 Pseudomonas pneumoniae<br>J152 Pneumonia due to staphylococci<br>J153 Pneumonia due to Streptococcus group B<br>J154 Pneumonia due to other streptococci<br>J155 Pneumonia due to Escherichia coli<br>J156 Pneumonia due to other Gram-negative bacteria<br>J158 Other bacterial pneumopathies<br>J159 Bacterial pneumonitis, unspecified

VARIABLE	ROLE	DATA source(s)	OPERATIONAL DEFINITION
			J168 Pneumonia due to other infectious micro-organisms<br>J180 Bronchopneumopathy, unspecified<br>J181 Lobar lung disease, unspecified<br>J188 Other pneumonitis, microorganism unspecified<br>J189 Pneumopathy, unspecified
Lymphomas or skin cancers	Secondary outcome	SNDS	All patients date with at least one hospital stay with one of the following ICD10 codes after index date:<br>C81 Hodgkin's lymphoma<br>C82 Follicular lymphoma<br>C83 Non-follicular lymphoma<br>C84 Mature T/NK cell lymphoma<br>C85 Non-Hodgkin's lymphoma, other and unspecified types<br>C86 Other specified types of T/NK cell lymphomas<br>C792 Secondary malignant skin tumour
Drug-induced interstitial lung disorders	Secondary outcome	SNDS	All patients date with at least one hospital stay with one of the following ICD10 codes after index date (DP/DR):<br>J704 Interstitial lung disease, drug-induced, unspecified
Multifocal leukoencephalopathies	Secondary outcome	SNDS	All patients date with at least one hospital stay with one of the following ICD10 codes after index date (DP/DR):<br>A812 Multifocal leukoencephalopathy
Thrombocytopenia requiring hostpitalization	Secondary outcome	SNDS	All patients date with at least one hospital stay with one of the following ICD10 codes after index date (DP/DR):<br>D695 Secondary thrombocytopenia<br>D696 Thrombocytopenia, unspecified
Neutropenia requiring hospitalization	Secondary outcome	SNDS	All patients date with at least one hospital stay with one of the following ICD10 codes after index date (DP/DR):<br>D70 Agranulocytosis<br>D611 Drug-induced bone marrow aplasia<br>D613 Medullary aplasia, idiopathic<br>D619 Medullary aplasia, unspecified
Anemia requiring hospitalization	Secondary outcome	SNDS	All patients date with at least one hospital stay with one of the following ICD10 codes after index date (DP/DR):<br>D50 Iron deficiency anemia

VARIABLE	ROLE	DATA source(s)	OPERATIONAL DEFINITION
			D62 Acute posthaemorrhagic anaemia<br>D630 Anemia in tumor diseases (C00-D48)<br>D638 Anemia in other chronic diseases classified elsewhere<br>D649 Anemia, unspecified
Cytopenia requiring hospitalization	Secondary outcome	SNDS	All patients date with at least one hospital stay with anemia, neutropenia and/or thrombocytopenia as defined in the 3 lines above.
Hypercholesterolemia requiring hospitalization following the initiation of sirolimus	Secondary outcome	SNDS	All patients date with at least one hospital stay with the following ICD10 codes after index date (DP/DR):<br>E780 - Essential hypercholesterolemia
Initiation of lipid-lowering therapies within 6 months after sirolimus initiation	Secondary outcome	SNDS	Initiation of lipid-lowering treatment (ATC C10) during the 6 months post sirolimus initiation
High blood pressure flare-ups	Secondary outcome	SNDS	All patients date with at least one hospital stay with one of the following ICD10 codes after index date (DP/DR):<br>I158 Other secondary hypertension<br>I159 Secondary hypertension, unspecified<br>I150 “Vasculo-renal hypertension<br>I10 Essential hypertension (primary)
Repiratory tract hemorrhage requiring hospitalizations	Secondary outcome	SNDS	All patients date with at least one hospital stay with one of the following ICD10 codes after index date (DP/DR):<br>R04 Respiratory tract hemorrhage
Time between LAM diagnosis and treatment initiation with sirolimus	Secondary outcomes	SNDS	For probable LAM and TSC patients:<br>- Time between the first occurrence of event confirming LAM/TSC and first delivery of sirolimus

Precision : in the SNDS, the classification used is the ICD-10 (and not ICD-10-CM).

Supplementary Table S3 - Distribution of the reasons for exclusion from population treated with sirolimus

**Exclusion criteria**	**Number of Patients excluded**	**% Patients excluded based on these criteria**
Patients followed-up for renal transplantation	745	64.1%
Cancer*	383	33.0%
Renal transplantation	330	28.4%
Hepatic or intestinal transplantation	127	10.9 %
Graft versus Host Disease	115	9.9%
Auto-immune diseases	87	7.5%
Pancreatic transplantation	23	2.0%
Cardiac transplantation	9	0.8%
Refractory vascular abnormalities*	2	0.2%
**Total number of patients excluded as considered treated with sirolimus for another reason**	1,162	64.6%
** Different periods were considered: for refractory vascular abnormalities: within the 6 months before or after index date; for Cancer: 1-year prior index date (instead of 8 years before index date and 2 months after index date for the other criteria).*		

Supplementary Table S4 – Baseline characteristics

Variable	Probable LAM	TSC
N	208	33
Median (Q1; Q3) age in years	45.0 (34.0; 58.5)	40.0 (28.0; 56.0)
**N (%) of patients with the comorbidities of interest at index date**		
Obesity with hospital stay or bariatric surgery	20 (9.6%)	4 (12.1%)
Antihypertensive treatment received	40 (19.2%)	7 (21.2%)
Hypertension	60 (28.9%)	10 (30.3%)
Diabetes	12 (5.8%)	2 (6.1%)
Dyslipidemia	7 (3.4%)	1 (3.0%)
Lipid-lowering treatments	12 (5.8%)	1 (3.0%)
Epilepsy	5 (2.4%)	4 (12.1%)

Supplementary Table S5 – Incidence and prevalence rates of LAM treated with sirolimus for 1 000 000 French adult women

	Incidence rate for 1 000 000 French adult women		Prevalence rate for 1 000 000 French adult women	
**Year**	**Probable LAM**	**Possible LAM**	**Probable LAM**	**Possible LAM**
**2014**	0,48	1,04	2,01	4,27
**2015**	0,70	1,70	2,55	5,77
**2016**	0,41	1,92	2,91	7,59
**2017**	0,55	2,16	3,37	9,57
**2018**	0,84	3,50	3,90	12,63
**2019**	1,20	3,41	4,90	15,59
**2020**	0,83	2,92	5,52	18,11
**2021**	0,93	3,20	6,32	20,75

Supplementary Figure S1 - Distribution of clinical manifestations observed in patients to be considered as probable LAM

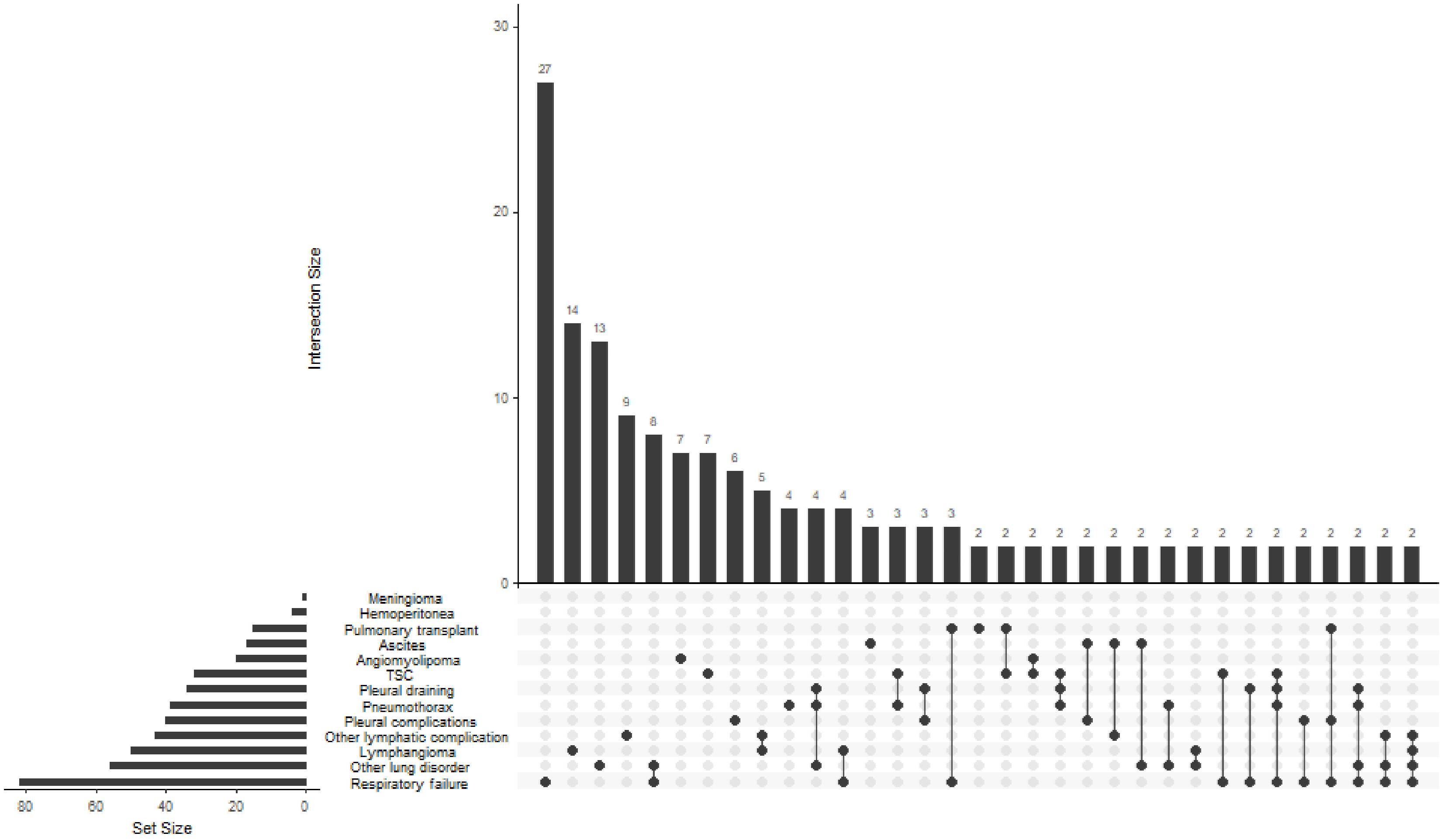

This intersection plot displays combinations of LAM clinical manifestations found in the probable LAM cohort. Each horizontal bars on the left side of the figure represents the number of patients who had experienced at least one LAM event listed next to the bar (for instance, a bit more than 80 patients had experienced respiratory failure, about 50 patients had a hospital stay for lymphangioma). The horizontal bars are ranked from the events involving the least patients (at the top) up to the events involving most patients (at the bottom). Each vertical bar represents the number of patients who had the considered combinations of events, which are ranked from combinations found in most patients on the left (i.e. 27 patients had only respiratory failure, 5 patients had an hospital stay for lymphangioma AND an hospital stay for another lymphatic complication) to the combinations the least found on the right. To keep readability, the graph has been truncated on the right and do not represent the integrality of combinations found in unique patients (N=67).

Supplementary Figure S2 - Time between sirolimus initiation and lung transplant observed in probable LAM patients (n= 208)

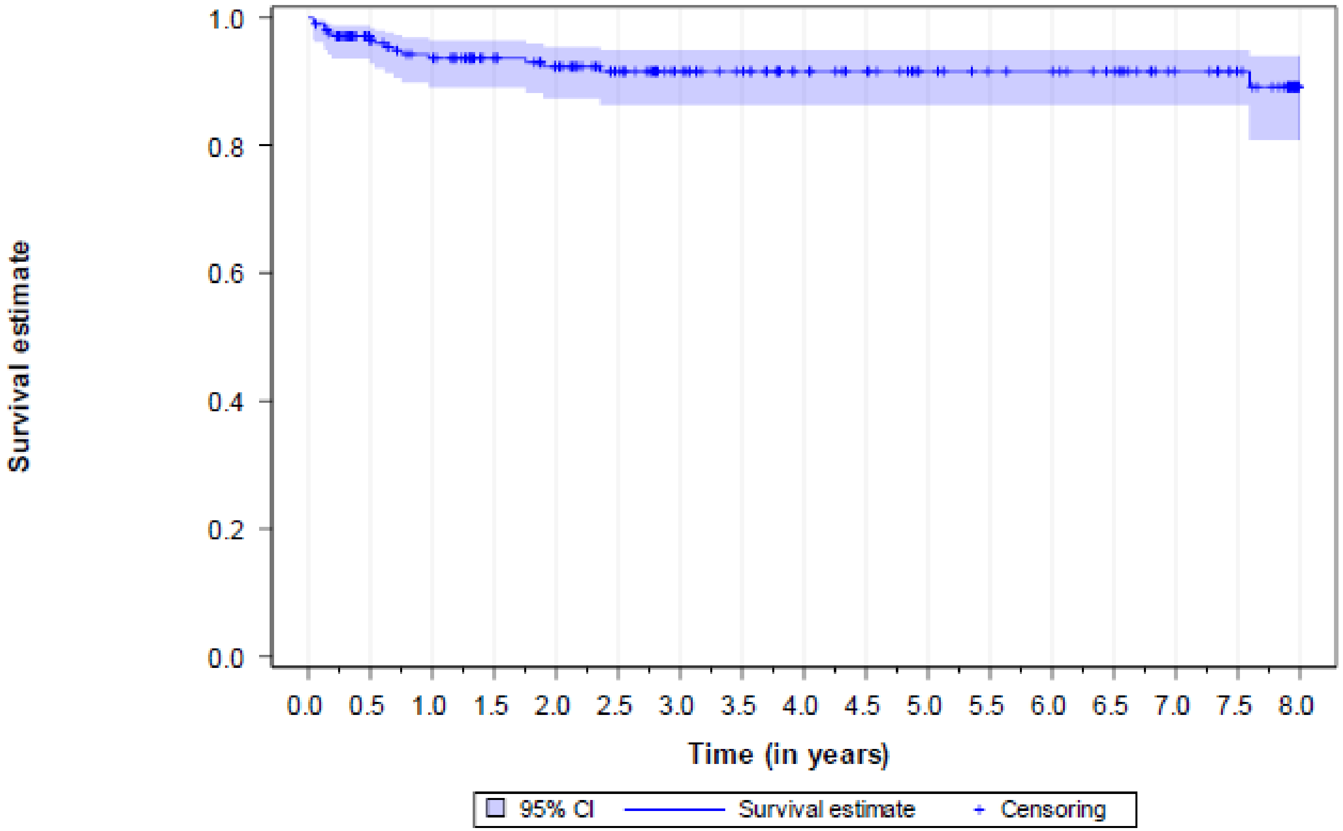

At risk	208	173	161	144	134	117	101	92	81	76	67	63	62	57	44	38	0
Cumulative events	0	7	12	12	14	15	15	15	15	15	15	15	15	15	15	15	16
Survival	1.00	0.97	0.94	0.94	0.92	0.92	0.92	0.92	0.92	0.92	0.92	0.92	0.92	0.92	0.92	0.92	0.89
